# When Waldenström macroglobulinemia hits the kidney: Description of a case series and management of a “rare in rare” scenario

**DOI:** 10.1002/cnr2.2062

**Published:** 2024-04-25

**Authors:** Nicolò Danesin, Greta Scapinello, Dorella Del Prete, Elena Naso, Tamara Berno, Andrea Visentin, Laura Bonaldi, Annalisa Martines, Roberta Bertorelle, Fabrizio Vianello, Carmela Gurrieri, Renato Zambello, Chiara Castellani, Marny Fedrigo, Stefania Rizzo, Annalisa Angelini, Livio Trentin, Francesco Piazza

**Affiliations:** ^1^ Hematology Unit, Department of Medicine University of Padova Padova Italy; ^2^ Nephrology, Dialysis and Transplantation Unit, Department of Medicine University of Padova Padova Italy; ^3^ Immunology and Molecular Oncology Diagnostic Unit, Veneto Institute of Oncology IOV‐IRCCS Padova Italy; ^4^ Veneto Institute of Molecular Medicine Fondazione per la Ricerca Biomedica Avanzata Padova Italy; ^5^ Cardiovascular Pathology Unit, Department of Cardiac, Thoracic and Vascular Sciences and Public Health University of Padova Padova Italy

**Keywords:** amyloidosis, kidney disease, lymphoma, lymphoproliferative disorders, Waldenström macroglobulinemia

## Abstract

**Background:**

Renal injury related to Waldenström macroglobulinemia (WM) occurs in approximately 3% of patients. Kidney biopsy is crucial to discriminate between distinct histopathological entities such as glomerular (amyloidotic and non‐amyloidotic), tubulo‐interstitial and non‐paraprotein mediated renal damage. In this context, disease characterization, management, relationship between renal, and hematological response have been poorly explored.

We collected clinical, genetic and laboratory data of seven cases of biopsy‐proven renal involvement by WM managed at our academic center and focused on three cases we judged paradigmatic discussing their histopathological patterns, clinical features, and therapeutic options.

**Case:**

In this illustrative case series, we confirm that serum creatinine levels and 24 h proteinuria are parameters that when altered should prompt the clinical suspicion of WM‐related renal involvement, even if at present there are not precise cut‐off levels recommending the execution of a renal biopsy. In our series AL Amyloidosis (*n* = 3/7) and tubulo‐interstitial infiltration by lymphoma cells (*n* = 3/7) were the two more represented entities. BTKi did not seem to improve renal function (Case 1), while bortezomib‐based regimens demonstrated a beneficial activity on the hematological and organ response, even when used as second‐line therapy after chemoimmunotherapy (Case 3) and also with coexistence of anti‐MAG neuropathy (Case 2). In case of poor response to bortezomib, standard chemoimmunotherapy (CIT), such as rituximab‐bendamustine, represents an effective option (Case 1, 6, and 7). In our series, CIT generates durable responses more frequently in cases with amyloidogenic renal damage (Case 1, 5, and 7).

**Conclusion:**

In this illustrative case series, we confirm that serum creatinine levels and 24 h proteinuria are parameters that when altered should prompt the clinical suspicion of WM‐related renal involvement, even if at present there are not precise cut‐off levels recommending the execution of a renal biopsy. Studies with higher numerosity are needed to better clarify the pathological and clinical features of renal involvement during WM and to determine the potential benefit of different therapeutic regimens according to the histopathological subtypes.

## 1 INTRODUCTION

Waldenström macroglobulinemia (WM) is a rare non‐hodgkin lymphoma characterized by bone marrow (BM) infiltration of neoplastic lymphoplasmacytic cells secreting a monoclonal IgM protein (mIgM).^1^ At variance with multiple myeloma, renal injury occurs rarely, in approximately 3% of WM patients.^2^, ^3^, ^4^ Renal involvement occurs also in about 6% of monoclonal gammopathy of undetermined significance (MGUS), thus resulting in monoclonal gammopathy of renal significance (MGRS).^5^ Organ damage may be caused by the mIgM directly, infiltration of tumor cells and by immune‐mediated mechanisms.^2^ Kidney biopsy is crucial to discriminate between the main forms of organ involvement such as glomerular, tubulo‐interstitial and non‐paraprotein mediated (Table 1).^2^, ^3^, ^6^ Glomerular disease in WM is classified in amyloidotic or non‐amyloidotic, and the latter can be related to cryoglobulinemic or non‐cryoglobulinemic membranoproliferative glomerulopathy, light and heavy chain deposition disease, or other causes (Table 1).^6^

**TABLE 1:** Spectrum of renal disease in Waldenström macroglobulinemia.

Glomerular amyloidotic	Glomerular non amyloidotic	Tubulointerstitial	Non paraprotein mediated
AL amyloidosis	Cryoglobulinemic membrane‐proliferative glomerulonephritis Non cryoglobulinemic membrane‐proliferative glomerulonephritis Light and heavy chain deposition disease Others	Cast nephropathy Lymphomatous infiltration Others	Minimal change disease Focal segmental glomerulosclerosis Thrombotic microangiopathy Membranous glomerulonephritis Acute tubular necrosis

AL amyloidosis accounts for the main cause of renal involvement in WM according to different series (Table 2).^2^, ^7^ Patients could present nephrotic syndrome and potential multiple organ involvement.^3^ Amyloid is defined as a misfolded protein able to self‐aggregate in extracellular fibrillar deposits. Monoclonal light or heavy immunoglobulin chains when aggregate in fibrillar structures may deposit and injure target organs. Glomerular damage in the kidney usually results in sub‐nephrotic or nephrotic proteinuria largely constituted by albumin and light chains (Bence–Jones proteinuria).^8^ Abdominal fat pad biopsy represents a first‐level diagnostic test. The gold standard test for demonstrating amyloid in the involved tissue remains the morphological assay on biopsied tissue with precise immunofluorescence features (clonality, Congo red positivity at apple green birefringence polarized light and thioflavin–t positivity at fluorescence microscope) and peculiar ultrastructural findings at electro‐immunomicroscopy (arranged nonbranching fibrils with positive immunogold staining for the subtype of amyloid deposits).^6^

**TABLE 2:** Clinical and molecular characteristics of patients at baseline.

	Case 1	Case 2	Case 3	Case 4	Case 5	Case 6	Case 7
Age at WM diagnosis, y	70	67	58	66	44	71	61
Renal BIOPSY diagnosis	AL amyloidosis	Membranoproliferative glomerulonephritis and LCDD	Lymphoma tubulointerstitial infiltration	AL amyloidosis	Lymphoma tubulo‐interstital infiltration	Lymphoma tubulo‐interstitial infiltration	AL amyloidosis^a^
IPSSWM, point	5	3	1	1	0	4	0
BM infiltration, %	100	3–4	65–70	80	70	100	100
CM, g L^−1^	47.9 + 6.2	2.1	23.6	26.2	4.46	17.4	6.2
Serum IgM, g L^−1^	55.3	3.06	25.9	35.1	7.3	24.2	8.3
Hb, g L^−1^	87	140	129	111	129	94	137
Albumin, g L^−1^	36	25	30	35	14.6	35	40
MYD88^L265P^	yes	yes	yes	Yes	Not tested	Not tested	Yes
CXCR4^S338X^	yes	no	no	Not tested	Not tested	Not tested	Not tested
Creatinine, μmol L^−1^ ^b^	117	402	231	86	60	146	157
Proteinuria, g/24 h^b^	2.98	21	0.40	3.6	25	2.03	0.6
Bence–jones proteinuria, g/24 h^b^	0.98	—	0.34	1.3	—	—	0.41
First line therapy (response)	DRC (PR)	BRD (PR)	BR (SD)	RM (PR)	DRC (CR)	BRD (PR)	B‐DRC (SD)
Second line therapy (response)	BR (MR)	—	BRD (PR)	BR (PR)	—	BRD (PD)	BR (PR)
Third line therapy (response)	Ibrutinib (PR)	—	—	—	—	BR (PR)	—

Abbreviations: B‐DRC, bortezomib, dexamethasone, rituximab, cyclophosphamide; BM, bone marrow; BR, bendamustine, rituximab; BRD, bortezomib, rituximab, dexamethasone; CM, monoclonal component; DRC, dexamethasone, rituximab, cyclophosphamide; Hb, hemoglobin; IPSSWM, international prognostic scoring system on Waldenstrom macroglobulinemia from Seventh International Workshop; LCDD, light chain deposition disease; MR, hematological minor response; PR, hematological partial response; RM, rituximab, melphalan; SD, hematological stable disease; WM, Waldenstrom macroglobulinemia.

^a^ Renal biopsy not already performed, diagnosis suggested by periumibical fat biopsy.

^b^ Highest level during the disease.

Non‐amyloidotic glomerular disease is considered another frequent type of WM‐mediated renal damage.^2^ Manifestations of non‐amyloidotic kidney disease could be hypertension, hematuria, or nephrotic‐range proteinuria.^8^ Type I cryoglobulinemia with systemic symptoms exacerbated with cold temperature and low serum levels of C4 complement factor can characterized cryoglobulinemic membranoproliferative glomerulopathy.^8^ The presence of IgM monoclonal paraprotein can involve capillary wall and occlude renal micro vessels contributing to glomerular damage. In case of Type II, cryoglobulinemia polyclonality can mask monoclonality making diagnosis at immunofluorescence more difficult.^6^ Light chain deposition disease (LCDD) and/or heavy chain deposition disease (HCDD) are features considered part of monoclonal immunoglobulin deposition disease (MIDD), a pattern of kidney damage more frequent in myeloma than in WM patients.^8^ Morphologically they are characterized by nodular mesangial sclerosis and tubular basement membrane thickening.^8^ Generally, clinical manifestations are decreased kidney function and sub‐nephrotic‐range proteinuria, which may evolve into renal insufficiency in case of delayed disease identification (Case 2).^8^ Tubulointerstitial disease is considered the third most frequent cause of renal damage in WM, usually presenting as acute kidney injury (AKI) (Case 3, 5, and 6).^3^ This is in line with results published by Törnroth et al., who collected data of 44 patients with interstitial malignant B‐cell infiltration, 87% of whom presented with acute renal insufficiency.^9^ Higgins et al in their large series from Mayo Clinic identified 14% of patients with tubulointerstitial disease with lymphoma infiltration representing the most prevalent etiology,^3^ It seems that AKI and tubular damage can be related to increased intrarenal pressure due to diffuse tubulointerstitial lymphoplasmacytic infiltrations.^3^ The main patho‐physiological entities belonging to tubulointerstitial disease are lymphoplasmacytic organ infiltration and more rarely cast nephropathy or Fanconi syndrome.^2^, ^3^

In this work, we describe our series of renal involvement in WM. We confirm similar characteristics with published series in terms of clinical features and histopathological damage patterns. We also discuss the different therapeutic strategies according to patient's characteristics and the possible correlation between renal and hematological response. The two largest series presented in literature did not provide details on the choice in first and subsequent lines of therapy.^2^, ^3^ As major limits, the retrospective nature of the study and the small numerosity highlight the necessity of designing prospective multicenter studies to better clarify the open issues that we have highlighted in this case series.

## 2 METHODS

All the selected cases received a diagnosis of WM according to the fifth WHO criteria and belonged to a single center cohort of 215 WM patients managed between 1990 and 2023 at the Hematology Unit of the Padua University Hospital, Italy.^1^

Patients affected by Waldenström macroglobulinemia with biopsy‐proven renal involvement were diagnosed throughout the natural course of the disease representing the 3.3% of the entire cohort. Clinical and biological data at baseline have been retrospectively collected and summarized in Table 2. We have decided to describe more in depth three of seven paradigmatic cases, considered representative of peculiar histopathological organ damages in WM. The remaining four cases present similar morphological features to Case 1, 2, and 3, and have also been more shortly described highlighting the sequence of therapy administered in each patient.

Histological, immunofluorescence and ultrastructural analyses on renal biopsies were performed at the Cardiovascular Pathology Unit of our institution. Physicians at the Dialysis and Transplantation Unit of the Padua University Hospital performed renal biopsies and contributed to the multidisciplinary management of the patients. A written informed consent was obtained by all patients and data managed according to the Declaration of Helsinki principles.

## 3 RESULTS

### 3.1 Case 1

**FIGURE 1:**
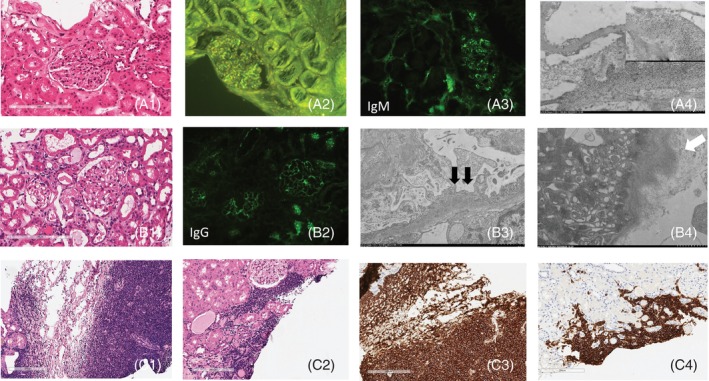
Histological, immunofluorescence, ultrastructural patterns in kidney biopsy of three WM patients with renal involvement. (A) Amyloidosis AL (Case 1): (A1) enlargement of the mesangium and thickening of the glomerular basal membranes due to amorphous deposits, hematoxylin‐eosin staining, scale bar 200 μm, original magnification 200×, (A2) thioflavin at fluorescence showing positivity for amyloid deposits original, original magnification 200×, (A3) immunofluorescence against IgM with granular positivity at glomerular basal membrane, original magnification 200×, and (A4) electron micrograph with immunoreactivity for one light chain of amyloid fibrilles as demonstrated by a post‐embedding immunogold method in keeping with light chain lambda amyloidosis (original magnification 10 000×, in the box a close up at 20 000×). (B) Membranoproliferative glomerulonephritis and light chain deposition disease (Case 2): (B1) diffuse homogeneous thickening of the glomerular basal membrane, hematoxylin‐eosin staining, scale bar 200 μm, original magnification 200×, (B2) immunofluorescence against IgG with granular positivity at glomerular basal membrane, original magnification 200×, (B3,4) electron micropgraphs with subendothelial deposits at the glomerular basal membrane (black arrow) and light chain deposits (white arrow) in the tubular basal membrane [original magnification (B3) 2500× and (B4) 5000×]. (C) Direct kidney lymphoplasmacytic infiltration (Case 3): (C1,2) dense subcapsular and tubulointerstitial infiltrates by neoplastic lymphocytes, hematoxylin‐eosin staining, scale bar 200 μm, original magnification 200×, (C3,4) immunochemistry for B cells (CD20 staining), original magnification 200×.

A 70‐year‐old man in followed‐up for a MGUS IgM/λ was then diagnosed with WM in 2013. The disease onset was characterized by acquired Von‐Willebrand disease and hyper‐viscosity syndrome that were treated with steroids and plasmapheresis. 24 h‐proteinuria was 0.8 g at that time. Bone marrow cytogenetics and molecular studies revealed a complex karyotype and the presence of MYD88^L265P^ and CXCR4^S338X^ mutations. Treatment with dexamethasone‐rituximab‐cyclophosphamide (DRC) was then started, obtaining a partial response after 8 cycles.^10^ In 2018, the patient underwent primary percutaneous coronary intervention for acute coronary syndrome and he was then started on acetyl salicylic acid 100 mg day^−1^. In 2020 the serum levels of the monoclonal component increased and anemia developed, therefore a second‐line therapy with rituximab‐bendamustine (BR) was administered for 5 cycles, however obtaining a minor response, thus a bruton tyrosine‐kinase inhibitor (BTKi) was initiated.^11^ After 10 weeks, despite achieving a hematological partial response, 24 h‐proteinuria progressively increased up to 2.9 g (Table 2). Periumbilical fat and renal biopsies were performed. Morphological, immunofluorescence and ultrastructural studies by means of post‐embedding immunogold analysis evidenced λ chain AL amyloidosis (Figure 1A). Cardiac and hepatic involvement was excluded. After 2 years of BTKi therapy and a good control of the disease a cerebral intraparenchymal hemorrhage occurred in the presence of a normal coagulation asset, which did not require a surgical treatment and did not lead to significant clinical sequelae. Aspirin and Ibrutinib were stopped. At 4 months after BTKi interruption, the patient is still in hematological partial response with a further unexpected decrease of the 24 h‐proteinuria below 1 g.

### 3.2 Case 2

A 66‐year‐old male was diagnosed in 2009 with MGUS IgM/κ and severe symptomatic anti‐myelin‐associated glycoprotein (MAG) neuropathy, for which he was treated with four weekly‐doses of rituximab.^12^

In the subsequent years, the renal function progressively declined and a chronic kidney disease lasted until 2022 when the patient was admitted to the nephrology unit and diagnosed as having nephrotic syndrome (24 h proteinuria of 8 g without BJ protein) and acute kidney injury on chronic kidney disease fifth stage (serum creatinine 402 μmol L^−1^). A bone marrow biopsy was performed confirming MYD88^L265P^ mutated WM. For the suspect of organ involvement, a kidney biopsy was executed revealing a non‐cryoglobulinemic membranoproliferative glomerulopathy and κ light chain deposition disease (LCDD). Immunofluorescence and electron‐immunomicroscopy studies confirmed the involvement of glomerular and tubular basal membranes (Figure 1B). Because of the persistent alteration of renal function and worsening neuropathy, a therapy with rituximab‐bortezomib‐dexamethasone (BRD) was started.^13^ The patient was able to tolerate all the five courses of therapy and a significant improvement of renal function was achieved (serum creatinine levels dropped from 402 to 110 μmol L^−1^ and 24 h proteinuria from 21 to 0.28 g), together with a maintained partial hematological response and improvement of the peripheral neuropathy.

Differently from proliferative glomerulonephritis with monoclonal immunoglobulin deposits (PGNMID), in Case 2 the described renal damage entities remained separate.^8^ Besides, LCDD is characterized by tubular basement membrane staining, which is missing in PGNMID (Figure 1).^8^

### 3.3 Case 3

The third patient was a 58‐year‐old male referred to our unit after the detection of elevated mIgM/κ serum levels (26.5 g L^−1^), a reduced renal function (creatinine serum level 200 μmol L^−1^) and 24 h Bence–Jones proteinuria of 0.34 g. A bone marrow biopsy was consistent with WM harboring normal karyotype and MYD88^L265P^ mutation. Imaging also revealed an extensive nodal involvement. 18‐FDG PET‐CT scan was not indicative of disease transformation. The persistent reduced renal function in the absence of a clear etiology mandated a renal biopsy procedure. Light and immunofluorescence microscopy and molecular studies were conclusive for a diagnosis of lymphoplasmacytic infiltration of tubulo‐interstitial and subcapsular space (Figure 1C). The patient was then treated with bendamustine rituximab reaching a stable disease but with only partial improvement in the renal function (creatinine serum levels of 175 μmol L^−1^) after 3 cycles. Consequently, a second line therapy with bortezomib rituximab dexamethasone was started, obtaining a very good partial response after 5 cycles with also a significant improvement of the renal function (24 h proteinuria 0.06 g with absence of BJ proteinuria, creatinine level in the range of normality).

### 3.4 Other cases

The other four cases were represented by two AL amyloidosis (one established only performing periumbilical fat pad biopsy) and two tubule‐interstitial lymphoplasmacytic infiltration.

### 3.5 Case 4

A female patient of 66 years of age was treated in first line with 6 cycles of rituximab and chlorambucil after developing a symptomatic WM that manifested with hyperviscosity. She maintained a hematological partial response for 10 years, when she suddenly developed AKI with consistent 24 h‐proteinuria (3.6 g/24 h with 1.3 g/24 h of albuminuria) and worsening anemia. A renal biopsy was performed leading to the diagnosis of AL amyloidosis, that was confirmed also by the analysis of abdominal fat pad aspiration. Cardiac and hepatic involvement was excluded. The patient was treated with 6 cycles of rituximab‐bendamustine reaching a partial response with 24 h‐proteinuria value of 2.6 g/24 h without detectable BJ protein.

### 3.6 Case 5

Male, 53‐year‐old patient, affected by MGUS IgM/κ with a 7 years‐long follow‐up, who was referred to the emergency room because of rapid increase of body weight, severe oedema and proteinuria of 25 g/24 h associated to marked serum hypoalbuminemia of 14 g L^−1^. Suspecting that the nephrotic syndrome was due to the lymphoproliferative disease bioptic procedures were made of the bone marrow that confirmed a symptomatic WM and of the kidney that showed a tubulo‐interstitial infiltration of lymphoplasmacytic cells. Six cycles of DRC were then administered, followed by the disappearance of the monoclonal component and a near to normal 24 h proteinuria (0.27 g/24 h).

### 3.7 Case 6

Patient is 71‐year‐old man that at the time of WM diagnosis presented a creatinine serum level of 146 μmol L^−1^ and 24 h proteinuria of 2.03 g. After 6 cycles of BDR the renal function slightly improved (creatinine serum level of 117 μmol L^−1^) and a partial hematological response was achieved that lasted for 6 years. The creatinine serum level then rapidly increased again up to 266 μmol L^−1^ but with a minimal increase of the serum mIgM and a renal involvement was then suspected. Therefore renal biopsy was made that demonstrated lymphoplasmacytic tubulo‐interstitial infiltration. Hence, 1 cycle of BDR was administered, however without improving hematological and renal function (creatinine serum level raised up to 450 μmol L^−1^). BR therapy was then started and after 4 cycles the renal function ameliorated (serum creatinine level of 383 μmol L^−1^) with obtainment of an hematological partial response.

### 3.8 Case 7

He is a 61‐year‐old man diagnosed with WM and a creatinine serum level of 157 μmol L^−1^ with 0.4 g/24 h Bence–Jones positive proteinuria. For initial screening a periumbilical fat pad biopsy was made that suggested AL amyloidosis while cardiac magnetic resonance and cardiac damage biomarkers were highly suspicious for cardiac involvement (TnI 48.9 ng L^−1^; NT‐BNP 5897 ng L^−1^). A first line regimen based on bortezomib in addition to DRC was then started without substantial hematological response after 4 cycles. In addition, the renal function worsened (206 μmol L^−1^ creatinine serum level) and therefore therapy was switched to BR that was administered for 6 cycles obtaining a partial hematological response and regaining a normal renal function.

## 4 DISCUSSION

We herein presented seven cases of biopsy‐proven WM renal involvement identified in our center between 1990 and 2023, which are paradigmatic of the most prevalent types of renal damage in WM, according to previously published series.^2^, ^3^, ^7^ Based on the frequencies of the observed histological entities that showed AL amyloidosis and tubulo‐interstitial lymphoma infiltration as being the most prevalent types of renal involvement, our series show data that are in line with the two largest retrospective series published.^2^, ^3^

Specific renal lesions, clinical features, and management implications are different for each case. In Case 1, first line chemoimmunotherapy led to a hematological response maintained for 7 years. During second line chemoimmunotherapy, WM status did not significantly improve while the 24 h proteinuria increased and a diagnosis of AL amyloidosis was made. A BTKi was started that led only to a hematological response. The coexistence of BTKi, cardioaspirin, and the likely increased risk of acquired coagulopathy due to renal protein loss may have contributed to cause cerebral hemorrhage. Unexpectedly, interruption of BTKi was followed by a reduction of 24 h proteinuria. This case highlights the importance of monitoring renal function in WM patients. In case an elevated albuminuria and Bence–Jones proteinuria are detected, a kidney biopsy is strongly recommended to confirm the presence of AL amyloidosis. Even in the absence of evidence from prospective trials, there is agreement that bortezomib‐based regimens should be utilized when a amyloidotic renal involvement by WM is present, as suggested by the experience in multiple myeloma.^14^ There is also evidence that commonly used chemoimmunotherapy regimens (such as BR or DRC) can produce a good hematological and renal response in this subset of patients, while BTKi are not recommended due to their toxicity, especially risk of arterial hypertension, which can contribute to worsen renal function, or because of the increased risk of bleeding.^6^, ^15^ Indeed, in the specific case of AL amyloidosis, we suggest to consider the increased risk of bleeding due to acquired coagulation disorders that would indicate the avoidance of BTKi.^16^ In this special case it is possible that the maintained hematological response after BTKi stopping could be a long effect of the previous CIT line.

The second patient described presented an infrequent type of organ involvement with the presence of both non‐cryoglobulinemic membranoproliferative glomerulopathy and light chain deposition disease, not classifiable as proliferative glomerulonephritis with monoclonal immunoglobulin deposits (PGNMID). As mentioned, bortezomib‐based regimens represents probably the most effective front‐line option in presence of WM‐related renal damage due to efficacy in hematological and renal response. In this case, the coexistence of anti‐MAG neuropathy represented a concern for the use of bortezomib. However, the patient did not experience a worsening of the symptoms but instead showed a neurological improvement, suggesting that also in this context bortezomib could be an effective therapeutic choice.

In the third case, where WM presented as AKI due to massive tubulo‐interstitial infiltration by lymphoplasmacytic cells, the use of the BR regimen was surprisingly rather ineffective. Instead, a good response was achieved employing a bortezomib‐based regimen, demonstrating that this therapy also represents a good choice in second‐line WM with kidney infiltration.

The last four cases showed again the variability of the clinical presentations and the difficulty to establish a standardized therapy sequencing. In this context, the role of CIT should be regarded as important, especially in those cases with demonstrated bortezomib inefficacy. Although there are no data in the literature to support the finding, it seems that the cases with AL amyloidosis of our series may have better responses when CIT regimens such as BR or DRC are used (Case 1, 4, and 7). How to combine bortezomib‐based regimens and CIT depends on the single patient conditions, comorbidities, and therapy tolerance. Analysis of larger retrospective or prospective series is needed to address the issue of whether bortezomib should better be used before or after CIT in the sequential therapy of such difficult condition. Indeed, to the best of our knowledge there is a lack of evidence on the best sequencing of therapy in case of renal involvement by WM. In the cited retrospective series, it has been described the outcome of the therapies utilized without deepening on the sequencing.^2^, ^3^

## 5 CONCLUSION

The illustrative case series here reported confirms the role of the strict monitoring of renal function (creatinine serum levels and 24 h proteinuria) to intercept potentially severe WM renal involvement, even if there are not precise cut‐off levels useful to recommend the biopsy procedure.^7^ From the therapeutic standpoint, in the absence of prospective trials, real life experience indicates that BTKi may not be an optimal front‐line choice to achieve fast improvement of renal function (Case 1). Instead, bortezomib‐based regimens seem to be efficacious to obtain good hematological and organ response, even after previous chemoimmunotherapy (Case 3).^6^ Because of the bortezomib‐related neurotoxicity, its use in patients with the coexistence of anti‐MAG neuropathy should be carefully weighted but still considered (Case 2).^12^, ^14^ In case of bortezomib inefficacy standard chemoimmunotherapy (CIT) regimen such as rituximab‐bendamustine represents an alternative (Case 6 and 7). Furthermore, limited to our data, CIT generates durable responses more frequently in amyloidogenic renal damage (Case 1, 5, and 7). Thus, the data from our little series suggest that bortezomib‐based regimens and CIT such as BR or DRC represent therapeutic options that need to be placed in first two lines of therapy when “WM hits the kidney.” Multicenter, prospective observational trials are needed to better clarify the pathological, clinical and management‐related features of this rare condition occurring in a rare blood disease. Lastly, considering the risks associated with biopsy and its unavailability in many centers, the identification of renal function tests, such as serum creatinine and/or 24 h‐proteinuria, with established cut‐off levels that discriminate those cases, for which a renal biopsy is required, would represent a useful way to ameliorate the management of WM patients with renal involvement.

## AUTHOR CONTRIBUTIONS

*Conceptualization*: Francesco Piazza and Nicolò Danesin. *Formal analysis*: Francesco Piazza, Nicolò Danesin, Greta Scapinello, Dorella Del Prete, Elena Naso, Chiara Castellani, Marny Fedrigo, Stefania Rizzo, and Annalisa Angelini. *Supervision*: Francesco Piazza and Livio Trentin. *Resources*: Tamara Berno, Andrea Visentin, Laura Bonaldi, Annalisa Martines, Roberta Bertorelle, Fabrizio Vianello, Carmela Gurrieri, and Renato Zambello.

## CONFLICT OF INTEREST STATEMENT

The authors have stated explicitly that there are no conflicts of interest in connection with this article.

## ETHIC STATEMENT

We declare that the work submitted to Cancer Reports has been done in accordance to “Wiley's Publication Ethics”.

## CONSENT FOR PUBLICATION

Written and verbal informed consent was was obtained from all the partecipants of the study.

## Data Availability

The data support the findings of this study are available from the corresponding author, Francesco Piazza, upon reasonable request.
